# Warfarin-Induced Calcification: Potential Prevention and Treatment Strategies

**DOI:** 10.31083/j.rcm2309322

**Published:** 2022-09-16

**Authors:** Xiaowu Wang, Langang Peng, Jipeng Ma, Liyun Zhang, Jincheng Liu

**Affiliations:** ^1^Cardiovascular Surgery, Xijing Hospital, Fourth Military Medical University, 710032 Xi'an, Shaanxi, China

**Keywords:** warfarin, anticoagulation, calcification, prevention

## Abstract

Warfarin is clinically used as the first choice for long-term anticoagulant therapy, and for the prevention of thromboembolic events. However, when used at low doses in the long term or high doses in the short term, warfarin treatment may result in tissue calcifications—such as calcifications in the coronary arteries, peripheral vascular system, blood vessels of patients with atrial fibrillation and chronic kidney disease, and vascular valves—and atherosclerotic plaque calcification. These warfarin-induced calcifications may affect cardiovascular function and exacerbate diseases such as diabetes and hypertension. Studies have shown that quercetin, osteoprotegerin, sclerosin, and sodium thiosulfate may alleviate these effects by interfering in the Wnt/β-catenin, TG2/β-catenin, Bone Morphogenetic Protein 2 (BMP2), and Eicosapentaenoic Acid/Matrix Metallopeptidase-9 (EPA/MMP-9) pathways, respectively. Nevertheless, the mechanism underlying warfarin-induced calcification remains unknown. Therefore, the question as to how to effectively attenuate the calcification induced by warfarin and ensure its anticoagulant effect remains an urgent clinical problem that needs to be resolved. To utilize warfarin rationally and to effectively attenuate the calcifications, we focused on the clinical phenomena, molecular mechanisms, and potential strategies to prevent calcification. Highlighting these aspects could provide new insights into the effective utilization of warfarin and the reduction of its associated calcification effects.

## 1. Introduction

Oral warfarin anticoagulation (OAC) administration is the main strategy for clinical anticoagulation, and effectively prevents various thromboembolic diseases. It is considered the first choice among long-term anticoagulant drugs for prevention of diseases, such as pulmonary embolism and deep vein thrombosis after mechanical heart valve replacement [1, 2]. However, one of the lesser-known long-term side effects of warfarin use is an increase in systemic arterial calcification [3]. Clinical and animal experimental data have demonstrated that long-term use of warfarin can lead to calcification of multiple tissues throughout the body [4, 5], leading to increased vascular wall stiffness and reduced compliance. These pathological side-effects may lead to serious complications, such as atherosclerosis, valvular calcification, and coronary artery calcification.

While the anticoagulant effect of warfarin is used extensively in clinical practice, treatment strategies addressing warfarin-induced calcification are still lacking. Previous studies have shown that quercetin, osteoprotegerin, sclerostin, and sodium thiosulfate can alleviate warfarin-induced calcification, mainly through the activity of Wnt/β-catenin [6, 7, 8], TG2/β-catenin [9, 10], Bone Morphogenetic Protein 2 (BMP2) [11, 12], and Eicosapentaenoic Acid/Matrix Metallopeptidase-9 (EPA/MMP-9) signaling pathways [13]. Nevertheless, the specific mechanism of action is unclear and there is no theoretical evidence to guide clinical practice. Therefore, warfarin-induced calcification is a significant clinical problem that needs to be urgently addressed. This review discusses the types of warfarin-induced vascular calcification (VC), proposes their potential mechanisms, and provides theoretical evidence for the rational use of warfarin for anticoagulation to reduce the calcification of tissues and its potential side effects.

## 2. Calcification Induced by Warfarin

### 2.1 Long-Term Use of Warfarin Can Induce Calcification of Small and Medium-Sized Arteries

#### 2.1.1 Calcification of Coronary Arteries Induced by Warfarin

Calcification of coronary arteries is a well-known risk factor for mortality in ischemic heart disease. Poterucha TJ *et al*. [14] demonstrated that the use of warfarin was associated with increased systemic calcification, including calcification of the coronary arteries and the surrounding vasculature. Andrews J *et al*. [15] evaluated the effects of warfarin on coronary percent atheroma volume (PAV) and calcium index (CaI), in patients with coronary heart disease. The results revealed that warfarin had no significant effect on PAV, but was independently correlated with increased CaI in a multivariate model. Namba *et al*. [16] assessed 42 patients with atrial fibrillation who had a high risk of developing atherosclerosis. The results revealed that long-term warfarin treatment may be related to osteoporosis and VC in hypertensive patients 60–80 years old. Villines *et al*. [17] conducted a cross-sectional analysis on the severity of coronary artery calcification (CAC) in patients without coronary heart disease treated with warfarin and found that the severity of CAC was positively correlated with the duration of warfarin use. Wei *et al*. [18] investigated the correlation between age and VC induced by warfarin. The data revealed that there was a dose-time-response for warfarin that was positively correlated with the distribution of the aortic calcification (AC) score and plasma IL-6 levels in patients less than 65 years old, but this correlation was not observed in patients ≥65 years old. Additionally, *in vitro* studies have demonstrated that warfarin treatment accelerates the calcification of vascular smooth muscle cells in young patients during the initial stages of calcification. The results suggest that aging and warfarin treatment are independently associated with increased AC. The sensitivity of warfarin-related AC in young patients is higher than that of elderly ones, which may be due to the increased cellular senescence induced by warfarin.

Animal experiments have also highlighted the effects of warfarin on AC. Uto *et al*. [19] investigated the role of collagen metabolism in AC. Male Sprague-Dawley rats (5 weeks old) were fed a diet containing warfarin and vitamin K1 (WVK) to establish a VC model; β-aminopropionitrile (BAPN) was utilized to inhibit lysyl oxidase (LOX), an enzyme that mediates collagen cross-linking. Transmission electron microscopy (TEM) and *in vitro* micro computerized tomography (μCT) showed that the extent of aortic medial calcification (AMC) in the rats that were fed a WVK diet increased with the duration of exposure.

#### 2.1.2 Calcification of the Peripheral Arteries Induced by Warfarin

Han *et al*. [20] assessed the incidence of peripheral AC in 430 patients treated with warfarin and found that warfarin was correlated with lower limb AC, but not with age, sex, diabetes status, or other characteristics. Using *in vivo* experiments, Mackay *et al*. [21] showed that mutations in the zebrafish (Danio rerio) lineal homologue *Abcc6a*, led to extensive and high mineralization of the axial skeleton, while warfarin aggravated its calcification phenotype, and vitamin K reduced ectopic calcification to normal levels.

#### 2.1.3 Calcification of Breast Arteries Induced by Warfarin

Tantisattamo *et al*. [22] found that warfarin administration increased the incidence of calcification of breast arteries in women. In a multivariate logistic model, warfarin was an important determinant of AC in women, and the severity of calcification was related to the age and duration of warfarin utilization, but not to the length of time after stopping warfarin treatment, indicating that warfarin-induced calcification of the breast arteries is cumulative and might be irreversible.

Breast fat necrosis (BFN) is usually considered a benign inflammatory response to breast trauma. AlQattan *et al*. [23] reported the case of a 65-year-old woman with atrial fibrillation who took warfarin. Examination of the histopathology revealed fat necrosis caused by calcification. Considering the background of the patient, the diagnosis was secondary BFN due to calcification induced by warfarin. Alappan *et al*. [24] investigated whether oral warfarin-induced VC could be reversed after renal transplantation and assessed the progression of calcification in the breast artery before and after renal transplantation. The data showed that VC is irreversible after renal transplantation, which highlighted the importance of prevention.

### 2.2 Vascular Calcification of Related Organs Induced by Long-Term Administration of Warfarin Can Aggravate Underlying Diseases

#### 2.2.1 Vascular Calcification Induced by Warfarin in Patients with Diabetes Mellitus and Hypertension

In fact, cardiovascular events are one of the major causes of deaths among patients affected with kidney disease and diabetes [25]. VC is a common complication in elderly patients with diabetes or renal insufficiency [26]. Warfarin was reported to cause vascular calcification, and renal arteries calcification with a decline in kidney function. As a result of kidney insufficient, most of the drugs used for cardiovascular risk reduction become unavailable [27]. Some patients with diabetes and hypertension need to take warfarin for an extended period of time, and some individuals need higher doses of warfarin to maintain a normal international normalized ratio (INR). However, long-term use of warfarin may lead to the calcification of small and medium-sized blood vessels and aggravate underlying diseases.

Zhang YT *et al*. [26] found that long-term warfarin treatment in patients with mechanical heart valve replacement, atrial fibrillation, hemodialysis, and chronic kidney disease could induce and accelerate VC, which not only leads to serious complications, such as atherosclerosis, valvular calcification, and CAC, but also aggravates diseases, such as diabetes and hypertension. Siltari *et al*. [28] revealed that warfarin increased the risk of further VC in patients with atherosclerosis. Bell DSH *et al*. [29] indicated that the incidence of non-valvular atrial fibrillation in patients with type II diabetes increased by 40%, and the risk of thromboembolism associated with atrial fibrillation increased by 79% compared with patients with atrial fibrillation without diabetes. Moreover, the use of warfarin in these patients improved thromboembolism, but decreased the level of matrix Gla protein, which may promote the calcification of the coronary and renal arteries, thus increasing the risk of cardiovascular disease and accelerating the decline of renal function. It has been reported that warfarin may accelerate hypertension in high-risk patients, especially in those with diabetes or uncontrolled hypertension [30].

#### 2.2.2 Vascular Calcification Induced by Warfarin in Patients with Atrial Fibrillation

Atrial fibrillation (AF) is a common complication in dialysis patients. Lee *et al*. [31] investigated the relationship between warfarin and congestive heart failure and peripheral arterial occlusive disease in AF patients on hemodialysis. The results revealed that warfarin-induced VC increased the risk of congestive heart failure and peripheral arterial occlusive disease in AF patients. Yamagishi *et al*. [32] evaluated the clinical efficacy and safety of warfarin use in patients with diabetes mellitus complicated with AF. Changes in blood glucose levels of diabetic patients may affect the pharmacokinetics and anticoagulant activity of warfarin, therefore the risk-benefit balance of warfarin may easily become impaired in these patients. Additionally, due to the vitamin K-dependent gamma-glutamyl carboxylation of warfarin inhibitors (Gla protein), the use of warfarin may increase the risk of osteoporotic fracture and VC, which are the main reasons for diminished quality of life in patients with diabetes complicated with AF. Brimble *et al*. [33] explored the relationship between end-stage renal disease (ESRD), AF, and the use of anticoagulants to prevent ischemic stroke. The data suggested that warfarin may not only increase the risk of bleeding, but also promote VC in this patient population.

#### 2.2.3 Vascular Calcification Induced by Warfarin in Patients with Chronic Nephropathy

Increased VC is “one of the main underlying mechanisms for cardiovascular death in patients with chronic kidney disease (CKD) mediated by cardiovascular disease (CVD)” [34, 35]. Clinical data has shown that warfarin is related to renal VC and the deterioration of renal function [36, 37]. Warfarin leads to the calcification of small and medium-sized arteries in patients with renal transplantation. Hristova *et al*. [38] reported that treatment with warfarin accelerated VC in patients who underwent renal transplantation, and that this was mainly noted in small and medium-sized arteries. On the contrary, there was almost no calcification in the aorta. Interestingly, calcification mainly occurred in the intima, indicating that the response to warfarin is different between the intima and media, and between the different vascular beds. In contrast to highly calcified renal vessels, renal allografts were not calcified.

Warfarin is one of the main factors associated with VC in patients with CKD and hemodialysis (HD) [39, 40]. Fusaro *et al*. [5] conducted epidemiological studies evaluating 387 hemodialysis patients that were followed for three years, to analyze the changes in mortality and the incidence of vertebral fracture and VC. In a multivariate logistic regression analysis, it was found that the use of warfarin was associated with an increased risk of aortic (OR 2.58, *p *< 0.001) and iliac artery calcification (OR 2.86, *p *< 0.001). During a follow-up period of 2.7 ± 0.5 years, 77 patients died, and patients treated with warfarin had a higher risk of death (HR 2.42, 95% CI 1.42–4.16, *p* = 0.001). Santos *et al*. [41] investigated the clinical characteristics and risk factors of death from calcified uremic atherosclerosis, and found that the use of warfarin may be a risk factor affecting disease progression in patients with CVD. Portales-Castillo *et al*. [42] reviewed how therapeutic drugs, including warfarin, affected the risk of calcification and related thrombosis, and found that warfarin was a key factor in the calcification process. Many clinical studies have shown that dialysis patients treated with warfarin have a higher risk of calcification than non-dialysis patients [43], and this effect was significantly enhanced in end-stage CKD [44]. Heaf *et al*. [45] noted that elderly patients using warfarin who received peritoneal dialysis were at an increased risk of VC. Böhm *et al*. [46] revealed a significant decrease in the glomerular filtration rate in patients with AF who received long-term anticoagulation treatment with warfarin. Fusaro *et al*. [47] reported that long-term use of proton pump inhibitors in HD patients aggravated VC. Hasegawa *et al*. [43] suggested that the use of warfarin in dialysis patients was a risk factor for skin necrosis and calcification, which was considered to be related to the transient hypercoagulable state or accelerated calcification induced by warfarin. Nigwekar *et al*. [37] investigated the risk factors of calcified uremia and revealed that warfarin treatment was associated with an increased risk for the development of calcific uremic arteriolopathy (CUA). The data derived from epidemiological studies and clinical investigations suggested that warfarin may lead to VC in patients with CKD.

### 2.3 Valvular Calcification Induced by Warfarin

Experimental data suggest that long-term use of warfarin can lead to valvular calcification. Levy *et al*. [48] observed the effects of vitamin K deficiency on mineral and bone metabolism. *In vitro* studies have shown that human aortic valve interstitial cells were calcified in the presence of high phosphate and a vitamin K antagonist. Using a rat model of calcific aortic valve disease (CAVD) induced by warfarin administration and vitamin K, Fang *et al*. [49] explored the role of miR-29b and TGF-β3 in vascular and valvular calcification. The data showed that inhibition of miR-29b in CAVD rats prevented vascular and valvular calcification and induced the expression of TGF-β3, suggesting that the miR-29b/TGF-β3 axis may play a regulatory role in the pathology of vascular and valvular calcification.

### 2.4 Calcification of Atherosclerotic Plaque Induced by Warfarin

Warfarin treatment has been shown to increase the volume of atherosclerotic plaques [50]. Van Gorp *et al*. [51] found that short-term treatment with warfarin promoted the formation of atherosclerotic plaques with a pro-inflammatory phenotype. Additionally, they found that warfarin aggravated the progression of plaque calcification and atherosclerotic disease. Long-term treatment with warfarin significantly accelerated the calcification of atherosclerotic plaques. Florea *et al*. [52] established a murine model of atherosclerosis using ApoE^-/-^ mice. After 12 weeks of warfarin administration, positron emission tomography/computed tomography was used to identify calcification in mice. The results showed that calcification in the warfarin group was significantly higher than that of the control group, especially in spotty calcifications at the proximal portion of the aorta.

### 2.5 Abnormal Calcification of Fetal Skeletal Cartilage Induced by Warfarin

Warfarin can induce fetal chondrodysplasia punctata (CDP), which is the abnormal calcification of skeletal cartilage during fetal development. CDP is usually inherited, but maternal vitamin K deficiency also leads to this particular pathology. Since warfarin is an oral anticoagulant that acts on vitamin K-dependent coagulation factors, the use of warfarin in pregnant women may facilitate the development of CDP. Therefore, warfarin is considered to be one of the non-genetic etiological factors associated with developing CDP [53]. Songmen *et al*. [54] reported that a 27-year-old woman who had taken warfarin after artificial heart valve surgery produced a fetus that developed warfarin syndrome. The baby was born with a sunken nasal bridge and narrow nostrils. X-ray images showed spotted osteophytes of the vertebrae, femur, and humerus, which supported the diagnosis of fetal warfarin syndrome. Between 1991 and 2007, Wainwright *et al*. [55] performed autopsies on 13 fetuses with warfarin embryonic disease, with a gestation period of 17 to 37 weeks, and among these fetuses, 11 cases had nasal dysplasia.

**Fig. 1.**
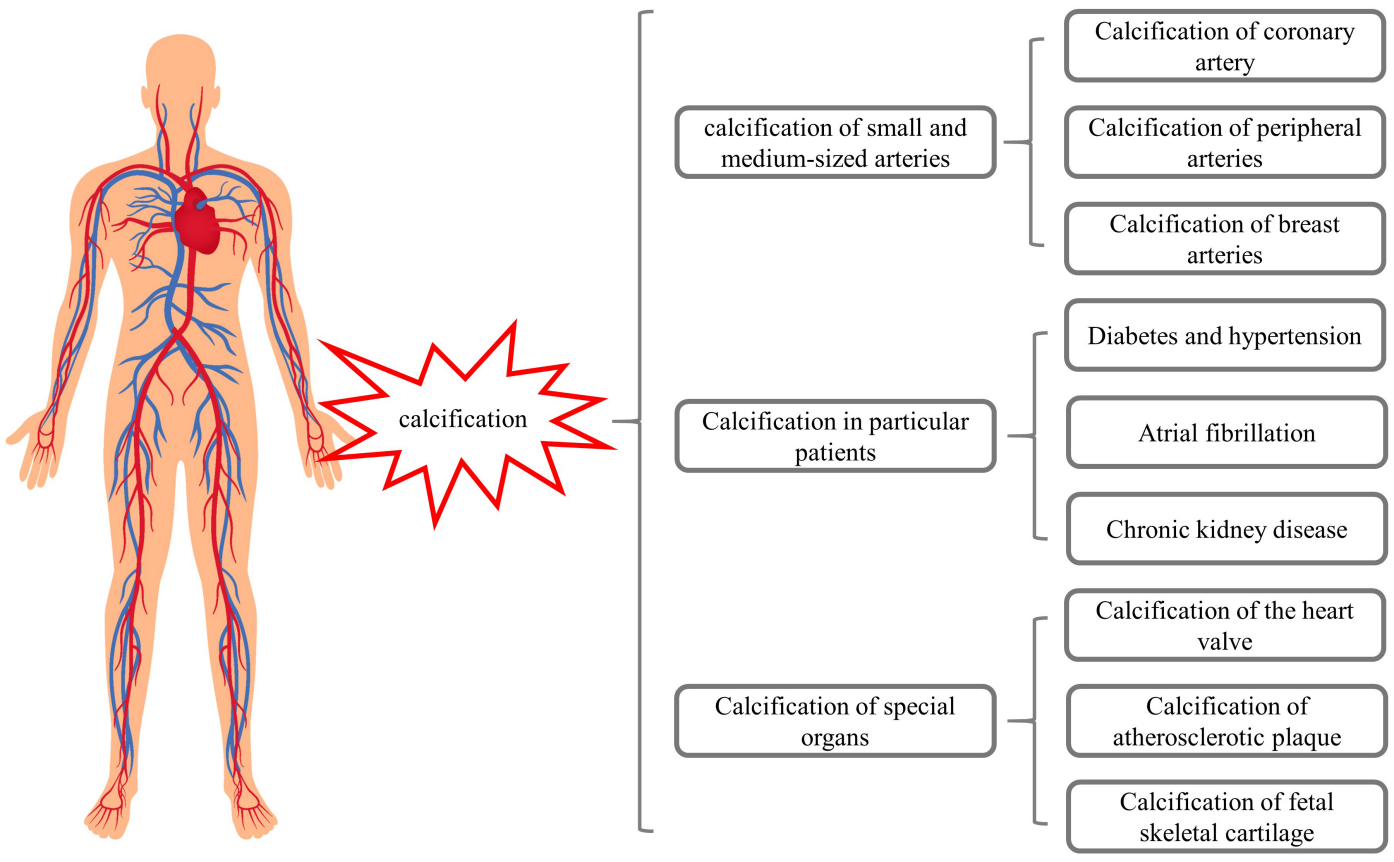
**Warfarin may induce calcifications of different organs**.

These studies suggest that warfarin may induce calcification of small and medium-sized arteries, breast arteries, and fetal skeletal cartilage (Fig. 1), while also promoting VC in patients with chronic diseases. These considerations should be highlighted in clinical practice when initiating warfarin treatment (Fig. 1).

## 3. Mechanisms Underlying the Calcification Induced by Warfarin

**Fig. 2.**
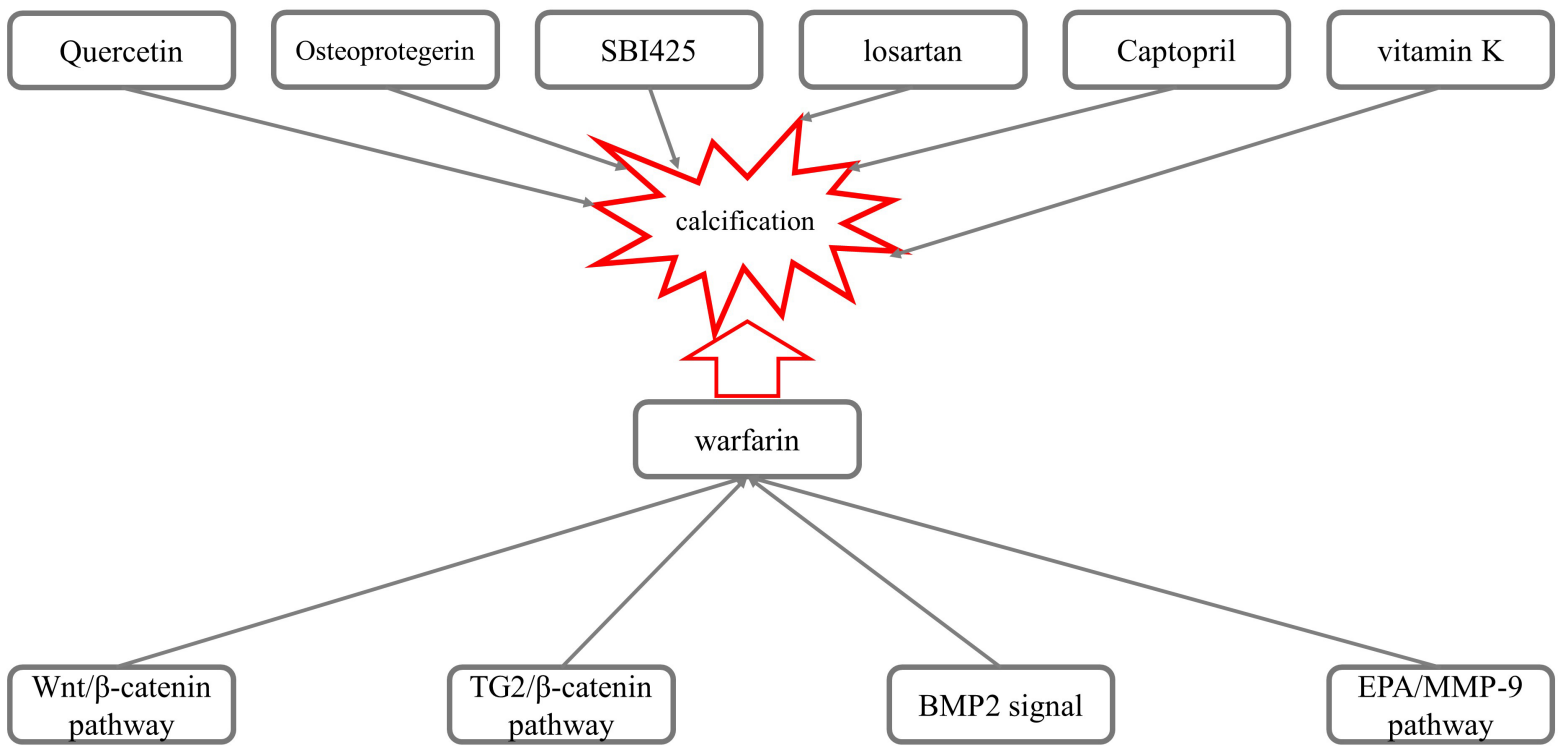
**Mechanisms underlying the calcification induced by warfarin**.

Warfarin-induced calcification is mainly due to the decreased synthesis and activity of matrix Gla protein (MGP). Gla is a vitamin K-dependent (VKD) amino acid that binds to calcium. It is mainly formed by post-translational modifications of glutamate induced by VKD γ-carboxylase. γ-carboxylation not only is an enzymatic process needed for vitamin K activation, but also involves other proteins that participate in bone formation and VC [26]. MGP is a VKD protein that prevents systemic calcification by scavenging calcium phosphate from tissues. Warfarin, which is a known vitamin K antagonist, is blocked by the synthesis and activity of MGP, and the inhibitory effect of MGP on VC is mediated by inhibiting the γ-carboxylation of MGP [56]. In addition, inhibiting the γ-carboxylation of MGP reduced the ability of GMP to bind calcium ions, leading to the deposition of calcium ions in blood vessels and other organs, promoting VC [56, 57, 58]. Warfarin treatment aggravated atherosclerotic plaque calcification with an increase in un-carboxylated MGP. Therefore, MGP may be considered a non-invasive biomarker of VC [59]. Researchers have found that MGP knockout mice had no other abnormalities except for being smaller in size compared with wild-type mice. However, all the mice died of arterial rupture by eight weeks after birth. Examination of their anatomy revealed extensive calcification of large and mid-sized arteries, calcification of elastic fibers in the media layer of blood vessels, loss of elasticity of vascular walls, and differentiation of smooth muscle cells into osteoblast-like (OBL) cells. This suggested that MGP acted as an effective VC protein [60, 61]. Warfarin mainly mediates calcification through the Wnt/β-catenin, TG2/β-catenin, BMP2 and EPA/MMP-9 signaling pathways (Fig. 2).

### 3.1 Wnt/β-Catenin Signaling in the Context of Warfarin-Induced Calcification

β-catenin is a bifunctional protein that regulates the coordination of cell adhesion and gene transcription. It is a subunit of the cadherin complex and acts as an intracellular signal transducer in the *Wingless* (Wnt) signaling pathway. Wnt signaling contributes to osteogenesis induction and activates downstream intracellular molecules by interacting with receptors on the cell membrane. This leads to the accumulation of β-catenin in the cytoplasm and facilitates its translocation into the nucleus. β-catenin has a wide range of biological functions [62, 63]. Cai *et al*. [6] found that the Wnt/β-catenin signaling pathway can directly regulate the expression of the *Runx2* gene in a high phosphorus environment and promote osteogenic differentiation of vascular smooth muscle cells (VSMCs). Bischoff *et al*. [64] found that targeting β-catenin could prevent VC induced by warfarin, and identified quercetin as a potential therapeutic drug for treating the calcification. Venardos *et al*. [65] found that warfarin induced osteogenic activity in normal and diseased isolated human aortic valve interstitial cell (AVICs). This effect is mediated by ERK1/2 in both diseased and normal AVICs, but in diseased AVICs β-catenin signaling also plays a role. These results implicate the role of warfarin in aortic valve calcification and highlight potential mechanisms for warfarin-induced aortic stenosis.

Nie *et al*. [7] investigated the role of the Wnt/β-Catenin pathway in medial arterial calcification. The results showed that warfarin aggravated the calcification of arteries and OBL cells by activating Wnt/β-catenin signaling. Beazley *et al*. [9] demonstrated that warfarin can mediate VC by inhibiting the formation of Gla, thus preventing MGP carboxylation. Activation of β-catenin signaling plays an important role in this process, indicating that the Wnt/β-catenin signaling pathway may be a novel target for the prevention of warfarin-induced VC. Quercetin (QU) is a frequently used drug that has a variety of biological activities, including cardiovascular protection. In the presence of QU, the activation of β-catenin by a glycogen synthase kinase-3β inhibitor reduced the accumulation of calcium on the vascular wall, which confirmed that the effects of QU were dependent on β-catenin inhibition. Further experiments showed that the inhibitory effect of QU was not involved in the induction of MGP carboxylation. The data revealed that down-regulation of MGP by shRNA did not change the effects of QU.

### 3.2 TG2/β-Catenin Signal Pathway and Calcification Induced by Warfarin

Several experimental results have suggested that the Transglutaminase 2 (TG2)/β-catenin signaling pathway plays an important role in the process of VC induced by warfarin. The results presented by Beazley *et al*. [9] showed that the β-catenin signaling pathway mediated by TG2 also plays an important role in VC induced by warfarin. It has been confirmed that warfarin-induced VC in rats is related to the accumulation and activation of TG2 and β-catenin signal transduction. Calcification induced by warfarin could be completely reversed by intraperitoneal injection of the TG2 specific inhibitor KCC-009 or dietary supplementation with flavonoid QU. This study showed for the first time that QU inhibited the activity of TG2. Moreover, QU stabilized the smooth muscle phenotype, prevented it from transforming into osteoblasts, reduced VC, and reversed the increased systolic blood pressure induced by warfarin. Studies performed by Beazley *et al*. [10] have shown that TG2 is a key mediator of warfarin-induced VC, and acts via activating β-catenin signal transduction in VSMCs. In addition, inhibition of the β-catenin pathway or TG2 activity reduced VC induced by warfarin. Therefore, it is suggested that the TG2/β-catenin signaling pathway may be a new target to prevent VC induced by warfarin.

### 3.3 BMP2 and Calcification Induced by Warfarin

BMP2 also participates in warfarin-mediated VC. Li *et al*. [11] observed the effect of losartan on warfarin-induced VC in rats. Compared with the control group, administration of losartan (100 ng/kg/day) for two weeks inhibited the expression of mRNA and the BMP2 and Runx2 proteins, as well as reduced the apoptosis of VSMCs and calcification induced by warfarin, suggesting that losartan inhibited VC via inhibiting the expression of Runx2 and BMP2. The results presented by Yu Z *et al*. [12] showed that warfarin accelerated the calcification of human aortic valve interstitial cells (HAVIC) in patients with aortic stenosis (AS) through the PXR-BMP2-ALP pathway.

### 3.4 EPA/MMP-9 Signal Pathway and Calcification Induced by Warfarin

The use of eicosapentaenoic acid (EPA) reduced the arterial calcification induced by warfarin in rats [13]. Sprague-Dawley rats were treated with warfarin (3 mg/g in food) and vitamin K1 (1.5 mg/g in food) for two weeks to induce medial arterial calcification, and then treated with EPA (1 g/kg/day). Immunohistochemical and RT-PCR analysis showed that EPA decreased the expression of osteopontin and osteogenic markers, such as alkaline phosphatase and core binding factor-α1, in the aorta. The migration of macrophages and the expression of matrix metalloproteinase (MMP)-2 or MMP-9 were observed around the calcifications of the aortic adventitia. EPA also reduced macrophage infiltration and the expression of MMP-9 and monocyte chemoattractant protein-1.

### 3.5 Other Mechanisms Underlying Warfarin-Induced Calcifications

Price *et al*. [66] revealed that osteoprotegerin effectively inhibited arterial calcification induced by warfarin. Compared to rats treated with warfarin alone, VC was significantly decreased in rats treated with both warfarin and osteoprotegerin. Osteoprotegerin completely prevented CAC induced by warfarin and reduced the levels of calcium and phosphate in the abdominal aorta (*p *< 0.001). These results suggested that osteoprotegerin can reduce warfarin-induced calcification, but the underlying mechanism is unclear.

Furmanik *et al*. [67] investigated the possibility that warfarin-induced aortic calcification and VC are primarily caused when endoplasmic reticulum stress increases the expression of Grp78 and ATF4 in rat aortas and VSMCs, increasing the release of extracellular vesicles through the PERK-ATF4 pathway and thus promoting VC. Opdebeeck *et al*. [68] found that administration of the tissue-nonspecific alkaline phosphatase (TNAP) inhibitor SBI-425 significantly reduced aortic and peripheral arterial calcification in a warfarin-induced calcification rat model. De Maré A *et al*. [69] used a diet containing warfarin to induce VC in rats to investigate the role of the bone formation inhibitor sclerosin (Sclerostin) in VC. The results showed that the severity of warfarin-induced VC was time-dependent, and the levels of serum sclerosin gradually increased.

## 4. Conclusions

Warfarin is widely used in patients who require long-term anticoagulation because of its effective anticoagulant properties, specific antagonism, and low cost. Presently, there is no ideal alternative drug [70, 71, 72]. More attention should be paid to keeping INR within a certain range to avoid bleeding or embolism when using warfarin. The dosage of warfarin is affected by many factors, such as sex, age, diet, and medication, and each patient should be dosed according to their individual needs and therapeutic goals [73]. However, the calcification induced by warfarin has not been highlighted by clinicians, as they prefer to focus on its anticoagulant effect. As such, there is no ideal strategy to prevent or treat warfarin-induced calcification. Therefore, reducing warfarin-induced calcification while ensuring the anticoagulant effect is an urgent clinical problem that needs to be resolved. It would be of great clinical significance to establish a scheme to reduce the calcification effects of warfarin.

### 4.1 Measures Based on the Mechanisms of Warfarin-Induced Calcification

OAC is a double-edged sword; on the one hand, warfarin exerts its anticoagulant effect by antagonizing vitamin K, on the other hand, it also induces VC by reducing the synthesis and activity of VKD MGP. Therefore, warfarin could not achieve a balance between anticoagulation and VC reduction by interfering with MGP. It has been confirmed that QU attenuates warfarin-induced VC via Gla/β-catenin or TG2/β-catenin signaling pathways. Losartan attenuated warfarin-induced VC by reducing the expression of Runx2 and BMP2, while osteoprotegerin reduced warfarin-induced calcification. Li *et al*. [74] established a rat model of arterial calcification with warfarin and vitamin K1. Two weeks after the induction of arterial calcification, rats were treated with captopril. The results revealed that the calcification of arteries was significantly attenuated after captopril treatment. Schurgers *et al*. [58] demonstrated that vitamin K could reduce warfarin-induced VC and reduce the decreased arterial distensibility that is induced by calcification. These data suggested that warfarin-induced VC could be alleviated by pharmacological intervention, but the related molecular mechanisms need to be further investigated.

### 4.2 Early Interventions and Multidisciplinary Treatment Strategies

Early clinical diagnosis, early intervention, and multidisciplinary approaches act in the prevention and treatment of VC induced by warfarin. Emamy *et al*. [75] reported that the direct factor Xa inhibitor slightly reversed calcification in coronary arteries and heart valves, which was induced by warfarin and other vitamin K inhibitors. Yang *et al*. [76] cultured HAVIC from patients with warfarin-induced aortic valve stenosis in a high inorganic phosphate medium, and showed that menaquinone-4 accelerated warfarin-induced calcification in HAVIC.

Some studies reported that warfarin-treated patients with supratherapeutic INRs had a much higher risk of adverse renal outcomes [77, 78, 79]. Clinicians need to make a trade-off between warfarin and new anticoagulant drugs, such as apixaban, for patients with AF on HD. On the one hand, warfarin has some shortcomings, such as inducing VC; on the other hand, anticoagulants such as apixaban or rivaroxaban have disadvantages, such as short half-life, lack of effective antagonism, and high cost, which limits their usability. Coleman CI *et al*. [34] compared the effects of rivaroxaban and warfarin on renal failure in patients with non-valvular atrial fibrillation (NVAF). Compared with warfarin, rivaroxaban reduced the incidence of renal failure. Reilly *et al*. [80] believed that apixaban is an ideal drug to replace warfarin in the treatment of HD complicated with NVAF. However, although both apixaban and rivaroxaban show good pharmacokinetic characteristics in ESRD, due to the potential risk of dialysis drug accumulation and the lack of adequate understanding of their mechanism, Brancaccio *et al*. [81] believed that neither of these two drugs could be used safely in dialysis patients. Saito *et al*. [82] reported five HD patients, four with medial arterial calcium deposits and one with skin calcium deposits; four patients were treated with sodium thiosulfate and three patients with low calcium dialysate. The average follow-up period was 7.4 months; however, four patients were cured and one died of infection. These data suggested that multidisciplinary, early management, and strict detection of minerals and bone markers may improve the process of warfarin-induced VC.

Warfarin-induced calcification is gradually becoming a concern for clinicians and some have tried to use newly developed drugs [3]. Still, all of these drugs have cumulative effects or lack definite antagonists, so they are unable to totally replace warfarin. Therefore, reducing calcification while ensuring the anticoagulant effect of warfarin is an urgent clinical problem that needs to be solved. The appropriate use of warfarin will be important for reducing calcification.

In this review, we summarize the clinical phenomenon of warfarin-induced calcification, its possible molecular mechanisms, and the current prevention and treatment strategies. We hope this review can provide a theoretical reference for further improvement of warfarin anticoagulation therapy, promotion of the rational use of warfarin anticoagulation, and minimization of its calcification effects.
